# T-bet and Eomes Are Differentially Linked to the Exhausted Phenotype of CD8+ T Cells in HIV Infection

**DOI:** 10.1371/journal.ppat.1004251

**Published:** 2014-07-17

**Authors:** Marcus Buggert, Johanna Tauriainen, Takuya Yamamoto, Juliet Frederiksen, Martin A. Ivarsson, Jakob Michaëlsson, Ole Lund, Bo Hejdeman, Marianne Jansson, Anders Sönnerborg, Richard A. Koup, Michael R. Betts, Annika C. Karlsson

**Affiliations:** 1 Division of Clinical Microbiology, Department of Laboratory Medicine, Karolinska Institutet, Stockholm, Sweden; 2 Immunology Laboratory, National Institutes of Health, Bethesda, Maryland, United States of America; 3 Center for Biological Sequence Analysis, Department of Systems Biology, Technical University of Denmark, Lyngby, Denmark; 4 Center for Infectious Medicine, Department of Medicine Huddinge, Karolinska Institutet, Stockholm, Sweden; 5 Department of Infectious Diseases Venhälsan, Stockholm South General Hospital (Södersjukhuset), Stockholm, Sweden; 6 Department of Laboratory Medicine, Lund University, Lund, Sweden; 7 Department of Microbiology, Tumor and Cell Biology, Karolinska Institutet, Stockholm, Sweden; 8 Unit of Infectious Diseases, Department of Medicine Huddinge, Karolinska Institutet, Karolinska University Hospital Huddinge, Stockholm, Sweden; 9 Department of Microbiology, University of Pennsylvania, Philadelphia, Pennsylvania, United States of America; University of Massachusetts Medical School, United States of America

## Abstract

CD8+ T cell exhaustion represents a major hallmark of chronic HIV infection. Two key transcription factors governing CD8+ T cell differentiation, T-bet and Eomesodermin (Eomes), have previously been shown in mice to differentially regulate T cell exhaustion in part through direct modulation of PD-1. Here, we examined the relationship between these transcription factors and the expression of several inhibitory receptors (PD-1, CD160, and 2B4), functional characteristics and memory differentiation of CD8+ T cells in chronic and treated HIV infection. The expression of PD-1, CD160, and 2B4 on total CD8+ T cells was elevated in chronically infected individuals and highly associated with a T-bet^dim^Eomes^hi^ expressional profile. Interestingly, both resting and activated HIV-specific CD8+ T cells in chronic infection were almost exclusively T-bet^dim^Eomes^hi^ cells, while CMV-specific CD8+ T cells displayed a balanced expression pattern of T-bet and Eomes. The T-bet^dim^Eomes^hi^ virus-specific CD8+ T cells did not show features of terminal differentiation, but rather a transitional memory phenotype with poor polyfunctional (effector) characteristics. The transitional and exhausted phenotype of HIV-specific CD8+ T cells was longitudinally related to persistent Eomes expression after antiretroviral therapy (ART) initiation. Strikingly, these characteristics remained stable up to 10 years after ART initiation. This study supports the concept that poor human viral-specific CD8+ T cell functionality is due to an inverse expression balance between T-bet and Eomes, which is not reversed despite long-term viral control through ART. These results aid to explain the inability of HIV-specific CD8+ T cells to control the viral replication post-ART cessation.

## Author Summary

CD8+ T cells display numerous traits of severe dysfunction in both treated and untreated HIV infection. Previous studies have demonstrated that HIV-specific CD8+ T cells in most individuals possess poor polyfunctionality, and an immature/skewed maturation phenotype. However, it remains unclear which transcriptional programming governs the regulation of CD8+ T cell differentiation and exhaustion in HIV infection. T-bet and Eomes represent two key transcription factors for CD8+ T cell differentiation and function, but surprisingly little is known about their influence of effector immunity following chronic viral infections in humans. In this study, we demonstrate that HIV-specific CD8+ T cells possess highly elevated levels of Eomes, but low T-bet expression. This differential relationship is linked to the up-regulation of several inhibitory receptors, impaired functional characteristics and a transitional memory differentiation phenotype for virus-specific CD8+ T cells. Importantly, these characteristics of HIV-specific CD8+ T cells remained stable despite suppressive ART for many years. These results implicate that reinvigoration of these cells might fail to elicit efficient responses to eradicate the viral reservoir.

## Introduction

An effective CD8+ T cell response is required to eradicate or control intracellular pathogens. During the acute phase of an infection, pathogen-specific CD8+ T cells expand and differentiate into effector cells to clear the microbe. In the wake of antigen clearance, long-lived memory CD8+ T cells develop in order to launch an effective secondary response against future infections. Murine studies have indicated that the process of memory formation is highly regulated by the T-box transcription factors T-bet and Eomesodermin (Eomes) [1], [2], [3]. Although T-bet and Eomes are related transcription factors that show some expressional overlap, their functional roles are not entirely reciprocal. Whereas T-bet regulates the expression of effector functions, Eomes is thought to primarily dictate the expression of proteins to maintain a memory CD8+ T cell repertoire that effectively could expand in case of re-infection [4], [5], [6], [7]. The current body of data thus suggest that the long-term fate of CD8+ T cell functionality and differentiation seems highly dictated by the expression ratio between T-bet or Eomes (reviewed in [8]).

Some viruses, including the human immunodeficiency virus type 1 (HIV), evade the immune defense and develop into chronic infection. As a consequence, the pool of HIV-specific CD8+ T cells persists throughout the infection and become dysfunctional. This process has usually been referred to as CD8+ T cell exhaustion, which is characterized by a typical loss of different functions, including the ability to proliferate, kill target cells (expression of cytotoxic molecules), and decreased IL-2, TNF, and IFNγ production [9], [10]. Initially, murine studies revealed that chronic lymphocytic choriomeningitis virus clone 13 (LCMV-13) infection caused an up-regulation of PD-1 [11] and other inhibitory receptors, like CD160, 2B4, and Lag-3, which cooperate to mediate CD8+ T cell dysfunction [12]. These findings were later extended to chronic human infections including HIV [13], [14], [15], [16], HCV [17], [18], [19], [20], and HBV [21], [22], [23]. In particular, HIV-specific CD8+ T cells have been studied with respect to dysfunctional characteristics, where seminal work have concluded that these cells in most individuals possess poor polyfunctionality [24], [25], and an immature/skewed maturation phenotype [26], [27]. However, it remains unclear which transcriptional programming that governs the regulation of CD8+ T cell differentiation and exhaustion in HIV infection.

Transcriptional networks have been related to CD8+ T cell exhaustion after LCMV-13 infection in mice [28], and recently also in HIV-specific CD8+ T cells specifically expressing PD-1 [29] and CD160 [30]. Lately, Wherry and colleagues have elucidated in the murine model that an exhausted profile following LCMV-13 infection is associated with an inverse relationship between T-bet and Eomes [31], [32]. Surprisingly, these studies showed that although T-bet caused terminal differentiation of CD8+ T cells, the transcription factor repressed expression of inhibitory receptors by direct binding to the promoter region of PD-1. Eomes on the other hand was highly associated with expression of numerous inhibitory receptors. In a recent study, long-term non-progressors were shown to retain high T-bet expression within HIV-specific CD8+ T cells [33]. However, it is currently unknown whether the expression levels of T-bet and Eomes in human virus-specific CD8+ T cells are associated with the up-regulation of inhibitory receptors, poor polyfunctionality, and the skewed maturation phenotype described in HIV-infected individuals.

To bring clarity to this, we examined the relationship between the expression of T-box transcription factors, markers of memory differentiation, and human viral-specific CD8+ T cell exhaustion at the single cell level. We provide evidence that HIV-specific CD8+ T cells in chronic infection largely possess highly elevated levels of Eomes, but lower T-bet expression. This relationship is associated with up-regulation of inhibitory receptors, impaired functional characteristics and a transitional memory differentiation phenotype. Importantly, these characteristics of HIV-specific CD8+ T cells remained stable despite suppressive ART for many years, and offer an explanation for the inability of CD8+ T cells to control viral replication post-ART cessation.

## Methods

### Ethics statement

The Regional Ethical Council (Stockholm, Sweden 2012/999-32 & 2009/1592-32) approved the study and all participants were provided with written and oral information about the study. Written informed consent was documented from all study subjects in accordance with the Declaration of Helsinki.

### Study participants

All HIV-infected individuals were recruited from the Karolinska University Hospital Huddinge and Venhälsan at Stockholm South General Hospital (Stockholm, Sweden). In total, 52 individuals with chronic untreated HIV infection and 12 HIV-infected individuals on ART for more than 10 years (fully suppressed viral load for >8 years) were enrolled in this study. Cell samples from 20 healthy controls were also collected from the Karolinska Institutet and Karolinska University Hospital Huddinge (Table 1). Out of the 52 individuals with chronic untreated HIV infection, 24 individuals were followed longitudinally from baseline (median = 0 days before ART initiation) and at 2 weeks, 4 weeks, 8 weeks, 12–16 weeks and 5–7 months post-ART initiation. All individuals initiated ART in chronic phase of infection and no one experienced virological failure (*i.e.* >200 HIV RNA copies/mL after 6 months on therapy) (Table S1).

**Table 1:** Cohort characteristics.

	HIV+ (ART−)^A^	HIV+ (ART+)^B^	Healthy controls
n	52	12	20
Age, years	38 (33–46)	48 (45–57)	37 (31–42)
Sex, n (%)	60% Males 40% Females	75% Males 25% Females	75% Males 25% Females
CD4 count, cells/µl	395 (245–550)	690 (590–940)	ND^C^
HIV RNA, copies/ml	28550 (3528–118500)	<50	NA^D^

Median (IQR) is shown for all parameters except n and sex.

A = Untreated HIV infected subjects.

B = Long-term treated HIV infected subjects.

C ND = Not Determined.

D NA = Not Applicable.

### Cells and peptides

Peripheral blood mononuclear cells (PBMCs) were isolated from whole blood by Hypaque-Ficoll (GE Healthcare) density gradient centrifugation and cryopreserved in FBS (Life Technologies) containing 10% DMSO. For detection of HIV- and CMV-specific T cell responses, peptide pools (15-mers overlapping by 11 amino acids) of HIV Gag-p55 (JPT technologies) and HCMV pp65 (NIH AIDS Research and Reference Reagent Program) were added to a final concentration of 1 µg/mL.

### Antibody reagents

All flow cytometry panels were tested on the HIV-infected and healthy control subjects within a 2-month time interval to avoid intra- and inter-individual differences of the flow analysis. The following antibodies were used: anti-CD3 APC-H7 (Clone SK7), anti-CD14 V500 (Clone M5E2), anti-CD19 V500 (Clone B43), anti-CD160 AF488 (Clone BY55), anti-CCR7 PE-Cy7 (Clone 3D12), anti-IFNγ AF700 (Clone B27), anti-TNF PE-Cy7 (Clone MAb11), anti-CD107a PE-CF594 (Clone H4A3), anti-HLA-DR BV605 (Clone G46) (BD Bioscience); anti-2B4 PE-Cy5.5 (Clone C1.7), anti-CD45RO ECD (clone UCHL1) (Beckman Coulter); anti-T-bet BV605 & -BV711 (clone B10), anti-CD4 BV650 & -BV785 (Clone OKT4), anti-PD-1 BV421 (clone EH12.2H7), anti-CD27 BV785 (clone O323), anti-CD38 APC (clone HIT2), anti-IL-2 BV605 (Clone MQ1-17H12), anti-Perforin BV421 (clone B-D48), anti-Granzyme A AF488 (clone CB9) (Biolegend); anti-T-bet PE (clone B10), anti-Eomes EF660 (clone WD1928) (eBioscience); anti-CD8 Qd565 (Clone 3B5), anti-CD4 PE-Cy5.5 (Clone S3.5), anti-Granzyme B PE-Cy5.5 (Clone GB11) (Life Technologies). LIVE/DEAD Aqua amine dye (Life Technologies) was used to discriminate dead cells and debris. MHC class-I tetramers and pentamers conjugated to PE (Beckman Coulter and ProImmune) were used to detect CD8+ T cells specific for HIV Gag SLYNTVATL (SL9)/HLA-A*0201, HIV Pol ILKEPVHGV (IV9)/HLA-A*0201 and CMV pp65 NVLPMVATV (NV9)/HLA-A*0201.

### Stimulation and intracellular staining of cells

PBMCs were thawed and washed twice in R10 (RPMI-1640 Medium AQmedia (Sigma Aldrich) containing 10% FBS, 50 IU/mL penicillin and 50 µg/mL streptomycin, 10 mM HEPES (Life Technologies)). Cells were counted on a Nucleocounter (ChemoMetec A/S), resuspended to 2×10^6^ cells/mL in R10 containing 10 U/mL DNase I (Roche Diagnostics) and rested for 5–6 hours at 37°C.

U-bottom plates were plated with 5 µg/mL Brefeldin A (Sigma Aldrich) and overlapping HIV Gag-p55 and HCMV pp65 peptides or medium alone (negative controls) together with 2×10^6^ PBMCs/well. When anti-CD107a was added at the start of the stimulation protocol [34], monensin (0.7 µg/mL, BD Bioscience) was also supplemented.

The PBMCs were next transferred to V-bottom plates, washed in PBS containing 2 mM EDTA and stained with a LIVE/DEAD Aqua amine dye solution containing the extracellular antibodies. Cells were first incubated for 10 minutes at 37°C with an anti-CCR7 antibody and then for further 20 minutes in room temperature with other extracellular antibodies. PBMCs were washed in PBS:EDTA and fixed and permeabilized using the FoxP3 transcription factor buffer kit (eBioscience). Monoclonal antibodies against T-bet, Eomes and other intracellular markers were incubated with the cells for 1 hour at room temperature in the dark. After further washing with Perm Wash solution (eBioscience), the PBMCs were resuspended in PBS containing 1% paraformaldehyde PFA. All specimens were acquired on the flow cytometer within the next 7 hours.

### Flow cytometric analyses

PBMCs were analyzed on a modified 4 laser LSR Fortessa (BD Biosciences). In total, approximately 1,000,000 events were collected per specimen. Antibody capture beads (BD Biosciences) were used to prepare individual compensation controls after separate stainings with all antibodies used in the experiments. FlowJo 8.8.7 (Treestar) was used for flow cytometric gating analyses. Most manual gatings were based on fluorescence minus one (FMO) gating strategies like previously described [35], [36]. A response was considered positive if the frequency of IFNγ producing cells were >0.05% of total CD8+ T cells after background reduction and twice the negative background.

### ELISA

Plasma was extracted from whole blood and stored in −80°C before analysis of soluble factors. Concentrations of cytokines in plasma were analyzed by ELISA using the following reagents: Ready-Set-Go, eBioscience (TNF, IL-6), Matched Antibody Pairs, eBioscience (IFNα) and HS Quantikine, RnD Systems (IL-12p70) according to the manufacturer's specifications.

### Statistics

Experimental variables between two groups of individuals were analyzed using Mann-Whitney U test and Wilcoxon matched-pairs rank test. Correlations were assessed using non-parametric Spearman rank tests. Bonferroni corrections were applied to all cases where multiple testing was performed. One-Way ANOVAs followed by Kruskal-Wallis non-parametric Dunn's multiple comparison tests were used to analyze three groups or more. The large data set consisting of functional and inhibitory receptor frequencies from the Boolean combinations was analysed by principal component analysis (PCA), an unsupervised statistical method for reducing data dimensionality while retaining the vital variation in fewer informative variables. The top 2 principle components (PCs) were plotted to visualize the trends such as clusters and outliers revealed by PCA. The Kolmogorov-Smirnov test was used to test the null hypothesis that the two groups of samples were drawn from the same distribution, where a large p-value suggests that the groups were drawn from the same distribution. All the statistical analyses were performed using GraphPad Prism 5.0 and R environment [37]. Permutation tests were analyzed using the data analysis program SPICE version 5.2009 [38].

## Results

### T-bet^dim^Eomes^hi^ CD8+ T cells express high levels of inhibitory receptors

**Figure 1:**
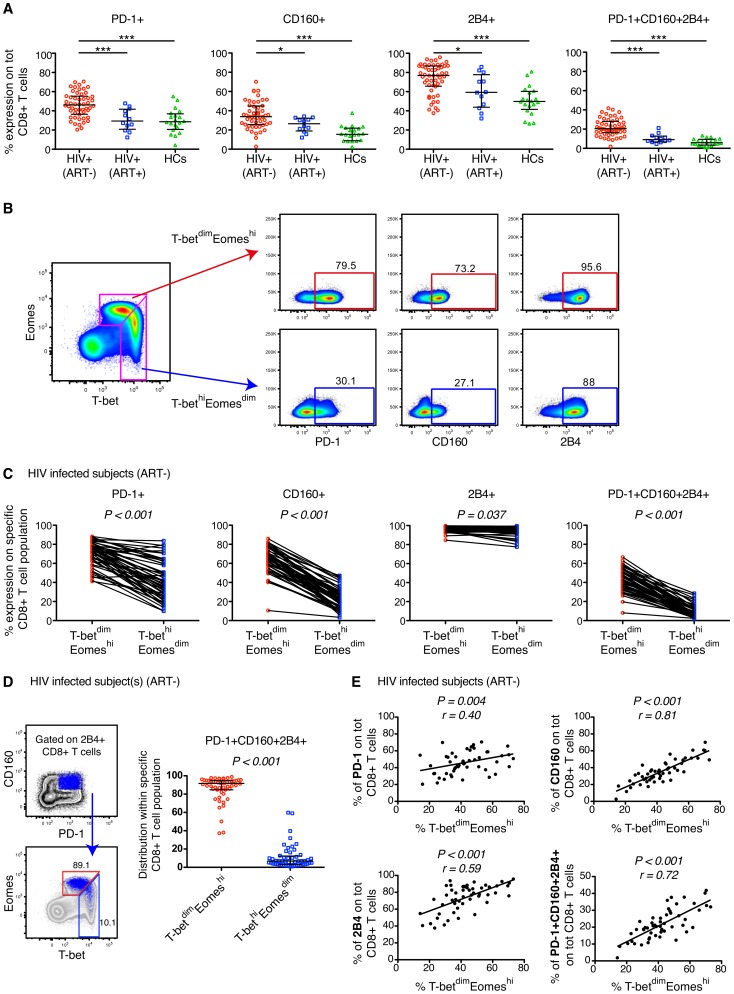
Expression patterns of inhibitory receptors and T-bet/Eomes on total CD8+ T cells. (A) The percentage expression of PD-1, CD160, 2B4 and co-expression of PD-1+CD160+2B4+ on total CD8+ T cells in chronic untreated HIV infected subjects, HIV+ (ART−) (n = 52); individuals on long-term ART, HIV+ (ART+) (n = 12); and healthy controls, HCs (n = 20). Statistical analysis was performed with one-way ANOVA, non-parametric Kruskal Wallis test with Dunn's multiple comparison test to compare all pairs of columns; *P<0.05, **P<0.01 and ***P<0.001. Median and IQR are depicted in the scatter plots. (B) Gating strategy to distinguish the T-bet^dim^Eomes^hi^ (red arrow) and T-bet^hi^Eomes^dim^ (blue arrow) memory population and subsequent expression patterns of PD-1, CD160 and 2B4 in a HIV infected subject. (C) The frequency of PD-1, CD160, 2B4 and PD-1+CD160+2B4+ on total T-bet^dim^Eomes^hi^ (red) and T-bet^hi^Eomes^dim^ (blue) CD8+ T cells for all untreated HIV infected subjects (n = 52). Statistical analysis was performed with Wilcoxon matched-pairs single rank test. (D) Gating strategy to distinguish the localization within the T-bet/Eomes axis of total PD-1+CD160+2B4+ CD8+ T cells for a HIV infected subject. The scatter plot shows the distribution of PD-1+CD160+2B4+ expression between T-bet^dim^Eomes^hi^ (red) and T-bet^hi^Eomes^dim^ (blue) CD8+ T cells. Wilcoxon matched-pairs single rank test was conducted to obtain P value. (E) Correlation analysis between the frequencies of T-bet^dim^Eomes^hi^ cells and PD-1, CD160, 2B4 and PD-1+CD160+2B4+ CD8+ T cells in all untreated HIV infected subjects. Spearman non-parametric test was used to test for correlations.

Previous studies have identified numerous inhibitory receptors, including PD-1, CD160, 2B4, LAG-3, CTLA4, and Tim-3, with increased expression patterns after chronic viral infections [13], [14], [15], [16], [39], [40]. Due to low and partly negligible expression of LAG-3, CTLA4 and Tim-3 on virus-specific CD8+ T cells (data not shown and [16]), we here focused on studying the expression of PD-1, CD160 and 2B4 on memory CD8+ T cells in HIV-infected individuals compared to healthy controls, and in relation to T-bet and Eomes expression. As expected, we found increased levels of CD8+ T cells mono- and co-expressing PD-1, CD160, and 2B4 in our cohort of 52 untreated HIV-infected individuals, compared to long-term treated (>10 years) HIV-infected subjects and healthy controls (Figure 1A and Figure S1A). However, no significant differences in frequency of CD8+ T cells expressing the inhibitory receptors was observed between the long-term treated HIV+ subjects and healthy controls (Figure 1A).

We next examined whether the expression pattern of T-bet and Eomes was linked to mono- and co-expression of PD-1, CD160, and 2B4 within the total CD8+ T cell population. A close relationship of increased expression of all inhibitory markers was observed for T-bet^dim^ and Eomes^hi^ CD8+ T cells (Figure S1B), thus corroborating murine studies of LCMV-specific CD8+ T cells [31]. We used a gating strategy similar to that recently described on CMV-specific CD8+ T cells [41], and gated on T-bet^hi^Eomes^dim^ and T-bet^dim^Eomes^hi^ CD8+ T cells (Figure 1B). In all untreated HIV-infected subjects, the T-bet^hi^Eomes^dim^ population contained significantly fewer cells with mono- and co-expression patterns of PD-1, CD160, and 2B4, as compared to T-bet^dim^Eomes^hi^ cells (Figure 1B–C). By gating on PD-1+CD160+2B4+ CD8+ T cells, we found that the vast majority (median = 91.7%) of these cells were T-bet^dim^Eomes^hi^ (Figure 1D). Further corroborating this link, we discovered that CD8+ T cells expressing inhibitory receptors was significantly associated with a T-bet^dim^Eomes^hi^ phenotype (Figure 1E). Notably, similar associations were also observed for individuals on long-term ART and matched healthy controls (Figure S1C). Together, these results indicate that an inverse expression pattern of T-bet and Eomes is highly associated with the up-regulation of several inhibitory receptors for total CD8+ T cells, independently of HIV infection status.

### Elevated expression of Eomes is a hallmark of HIV-specific CD8+ T cells

**Figure 2:**
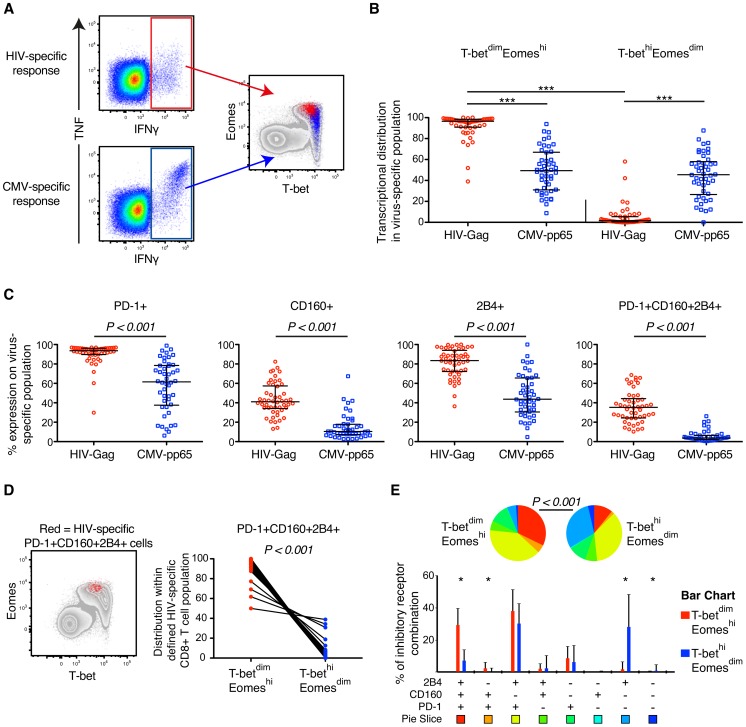
HIV- and CMV-specific CD8+ T cell expression of inhibitory receptors and T-bet/Eomes in untreated HIV infection. (A) Gating strategy illustrating the localization within the T-bet/Eomes axis of HIV- (red) and CMV-specific CD8+ T cells (blue). (B) Distribution within the T-bet^dim^Eomes^hi^ and T-bet^hi^Eomes^dim^ population of HIV- and CMV-specific CD8+ T cell responses. All data is derived from the untreated HIV infected subjects (n = 52). Median and IQR are shown in all graphs and non-parametric Kruskal Wallis test followed by Dunn's multiple comparison test was performed to compare all pairs of columns; *P<0.05, **P<0.01 and ***P<0.001. (C) The frequency of PD-1, CD160, 2B4 and PD-1+CD160+2B4+ on HIV- and CMV-specific CD8+ T cells. Mann-Whitney tests were performed to conclude significance between the groups (median and IQR). (D) Localization of PD-1+CD160+2B4+ expressing HIV-specific CD8+ T cells (red) within the T-bet/Eomes axis and distribution between the T-bet^dim^Eomes^hi^ and T-bet^hi^Eomes^dim^ population. Wilcoxon matched-pairs single rank test was performed to show on significant differences between the groups. (E) SPICE analysis of inhibitory receptors combinations between the T-bet^dim^Eomes^hi^ (red) and T-bet^hi^Eomes^dim^ (blue) population for HIV-specific CD8+ T cells. Median and IQR are shown for all bars and Wilcoxon matched-pairs single rank tests were performed to compare outcomes between groups; *P<0.05. Permutation test was performed between the pie charts.

As bulk CD8+ T cell expression of PD-1, CD160, and 2B4 was highly correlated with the T-bet^dim^Eomes^hi^ population, we next examined whether this relationship could be attributed to HIV- and other human viral-specific CD8+ T cells in general. We therefore stimulated PBMCs with overlapping peptide pools spanning HIV Gag-p55 and HCMV-pp65 to identify IFNγ/TNF-producing HIV- and CMV-specific CD8+ T cells, respectively [36], [42]. CMV-specific CD8+ T cell responses were selected as a control, since these responses have previously been shown to be highly polyfunctional and express less inhibitory receptors [16], [43], [44]. In Figure 2A, an example is shown for typical HIV- and CMV-specific IFNγ+/TNF+ responses. Notably, HIV-specific CD8+ T cells (n = 50) were almost exclusively T-bet^dim^Eomes^hi^, while CMV-specific CD8+ T cells (n = 46) could be detected in both the T-bet^dim^Eomes^hi^ and T-bet^hi^Eomes^dim^ populations in all untreated HIV-infected individuals with chronic disease (Figure 2A–B). The median fluorescence intensity (MFI) of Eomes and T-bet, was also significantly different between HIV- and CMV-specific CD8+ T cells (Figure S2A). Consistently, both the frequency and MFI of single and co-expression of PD-1, CD160, and 2B4 were elevated on HIV-specific compared to CMV-specific CD8+ T cells (Figure 2C and Figure S2B). We further gated on PD-1+CD160+2B4+ HIV-specific CD8+ T cells and found that this population almost exclusively were occupied within the T-bet^dim^Eomes^hi^ population (Figure 2D). By employing a SPICE analysis on HIV-specific CD8+ T cells, we further confirmed that co-expression of the inhibitory receptors were significantly elevated in the T-bet^dim^Eomes^hi^ population compared to T-bet^hi^Eomes^dim^ cells (P<0.001; Figure 2E).

**Figure 3:**
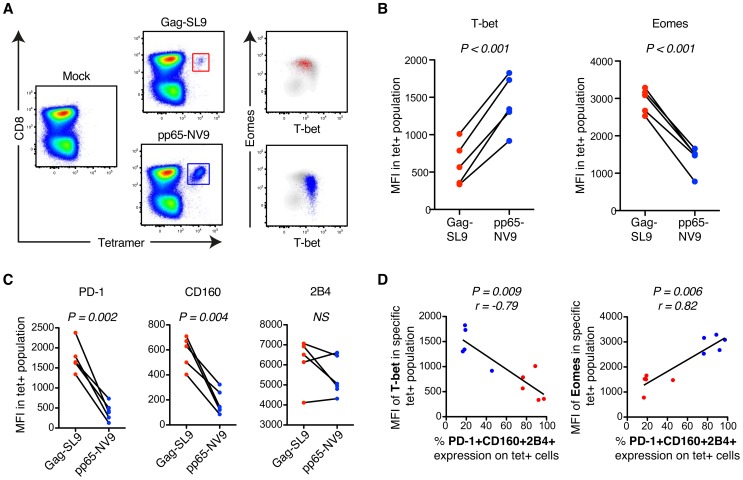
MHC class-I tetramer stainings of HIV- and CMV-specific CD8+ T cells in chronic HIV infection. (A) Gating strategy of HIV-Gag/SL9-tet+ (red) and CMV-pp65/NV9-tet+ (blue) CD8+ T cells and their distribution within the T-bet/Eomes axis. (B) The MFI of T-bet and Eomes for Gag/SL9-tet+ and pp65/NV9-tet+ cells. Paired t-tests were used to compare differences between the groups. (C) The MFI of PD-1, CD160 and 2B4 between Gag/SL9-tet+ and pp65/NV9-tet+ cells. Paired t-tests were used to compare differences between the groups. (D) Correlation analysis between the frequency of virus-specific PD-1+CD160+2B4+ cells and MFI of T-bet or Eomes. Spearman non-parametric test was used to test for correlations.

In further analysis we compared the relationship between expression of T-bet, Eomes, and inhibitory receptors among unmanipulated (resting) virus-specific CD8+ T cells. To this end, we directly examined HIV Gag SLYNTVATL (SL9) and CMV pp65 NVLPMVATV (NV9) specific CD8+ T cells in 5 HLA-A*0201+ donors, identified previously [45], by HLA class-I tetramer (tet) analysis. In agreement with our previous results, the HIV/SL9-tet+ cells had significantly higher levels of Eomes, but lower T-bet expression than CMV/NV9-tet+ cells (Figure 3A–B). The frequencies of CD8+ T cells mono- and co-expressing PD-1, CD160, and 2B4 were highly elevated among HIV/SL9-tet+ cells, compared to CMV/NV9-tet+ T cells (Figure 3C). Combining both HIV/SL9-tet+ and CMV/NV9-tet+ cells in one correlation analysis, we found an inverse correlation (P = 0.009, r = −0.79) between the MFI of T-bet and PD-1+CD160+2B4+ co-expression (Figure 3D). Similarly, a positive strong association (P = 0.006, r = 0.82) was observed between the MFI of Eomes and PD-1+CD160+2B4+ co-expression (Figure 3D). Together, these results show that human virus-specific CD8+ T cell regulation of inhibitory receptors is associated with high expresison of Eomes, but low T-bet expression.

### Limited virus-specific CD8+ T cell functionality is related to co-expression of inhibitory receptors and T-bet^dim^Eomes^hi^ cells

**Figure 4:**
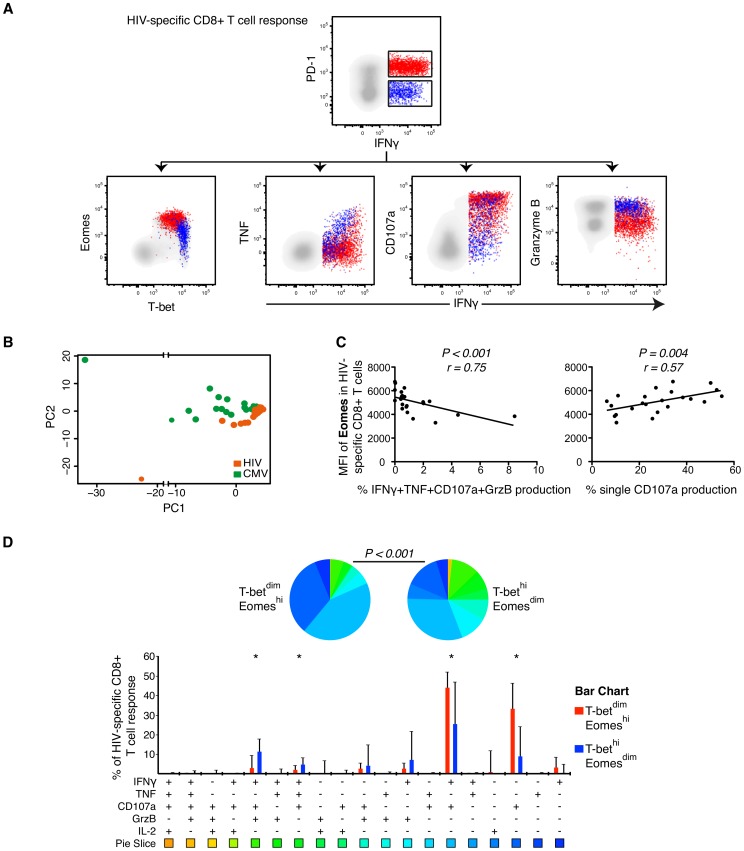
Polyfunctional characterization of virus-specific CD8+ T cells in untreated HIV-infected individuals. (A) Distribution of PD-1+ (red) and PD-1− (blue) HIV-specific CD8+ T cells within the T-bet/Eomes axis and linkage to IFNγ and TNF, CD107a and Granzyme B expression. (B) Boolean combinations of all functional and inhibitory markers (n = 256) were combined using PCA, generating a HIV- (orange) and CMV-specific (green) PC1 and PC2 score for each HIV infected subject (n = 23). (C) Correlations between the MFI of Eomes and IFNγ, TNF, CD107a and Granzyme B or single CD107a secretion. (D) SPICE analysis of all functional combinations between the T-bet^dim^Eomes^hi^ (red) and T-bet^hi^Eomes^dim^ (blue) population for HIV-specific CD8+ T cells. Median and IQR are shown for all bars and Wilcoxon matched-pairs single rank tests were performed to compare outcomes between groups; *P<0.05. Functional combinations where the IQR did not exceed 1% are not depicted in the graph. Permutation test was performed between the pie charts.

Co-expression of PD-1, CD160, and 2B4 has been closely linked to limited T cell functionality in mice and humans [12], [16]. Therefore, we next examined how differential expression of T-bet and Eomes was related to the functional properties of HIV- and CMV-specific CD8+ T cells in the context of these inhibitory markers. We assessed the expression pattern for IFNγ, TNF, IL-2, CD107a and Granzyme B simultaneously for both HIV- and CMV-specific CD8+ T cell responses in 23 untreated individuals with chronic HIV infection. All individuals generated responses, except one with lack of an anti-CMV response. PD-1^+^ HIV-specific IFNγ+ CD8+ T cells were found to be T-bet^dim^Eomes^hi^, whereas PD-1^−^ HIV-specific IFNγ+ CD8+ T cells were T-bet^hi^Eomes^dim^ (Figure 4A). The PD-1^+^ T cell response showed low co-expression of IFNγ with either TNF or Granzyme B. Interestingly however, we found that expression of CD107a together with IFNγ was more substantial in PD-1^+^ cells compared to PD-1^−^ cells. PD-1^−^ cells were more likely to simultaneously express IFNγ and TNF or Granzyme B together with CD107a.

Using multi-parametric flow cytometry analysis, we combined the measurment of all inhibitory receptors (PD-1, CD160, 2B4) together with the functional markers (IFNγ, TNF, IL-2, CD107a, Granzyme B) using Boolean gating principles (n = 256 T cell populations). The populations that were occupied in any HIV-infected subjects (n = 200) were further assessed using PCA analysis to determine whether HIV- and CMV-specific CD8+ T cells could be differentiated based on the combination of these markers (Figure 4B). A significant separation between the HIV- and CMV-specific responses was observed in terms of the PC1 and PC2 dimensions (Figure 4B) and confirmed by a Kolmogorov-Smirnov (KS) test, like previously described [46]. The KS test determines the probability that the two groups were drawn from the same distribution, where a P-value of 1 signifies that the groups were drawn from the same distribution. Particularly, the distributions of the PC2 scores for the two groups were shown to be derived from different distributions (D = 0.60, P<0.001). Additionally, the PC1 scores also showed significant differences between the groups (D = 0.46, P = 0.01).

We next evaluated specific correlations between functional markers and the T-bet/Eomes axis. The MFI of Eomes in HIV-specific CD8+ T cells was negatively associated with the co-expression of IFNγ, TNF, CD107a and Granzyme B (P<0.001, r = −0.72) and positively associated with single CD107a production (P = 0.004, r = 0.57; Figure 4C). The MFI of T-bet in HIV-specific CD8+ T cells was primarily correlated to co-expression of IFNγ, TNF and Granzyme B (P = 0.011, r = 0.52) (data not shown). Next, we assessed whether specific functional combinations were differentially distributed between the T-bet^dim^Eomes^hi^ and T-bet^hi^Eomes^dim^ HIV-specific CD8+ T cells by SPICE analysis. These analyses confirmed a polyfunctional diversity between the groups (P<0.001, permutation test) and that single production of CD107a (P<0.001), or together with IFNγ (P = 0.012), was elevated for T-bet^dim^Eomes^hi^ cells. In contrast, co-production of IFNγ, TNF and CD107a (P = 0.027) and IFNγ, CD107a and Granzyme B (P = 0.005) was expressed at higher frequencies by T-bet^hi^Eomes^dim^ cells (Figure 4D). We found similar associations for CMV-specific CD8+ T cells, showing that T-bet^dim^Eomes^hi^ cells particularly expressed high single production of CD107a, or together with IFNγ, while diverse combinations of all cytokines/cytotoxins were elevated within the T-bet^hi^Eomes^dim^ population (Figure S3). Altogether, our data suggests that increased expression of Eomes is linked to a profile of increased exhaustion for both HIV- and CMV-specific CD8+ T cells.

In order to further assess the link between T-bet and Eomes with cytolytic functions, we recruited 5 HLA-A*0201+ donors with identified HIV Pol ILKEPVHGV (IV9)-, HIV/SL9-, and CMV/NV9-specific CD8+ T cell responses. The effector functions were examined using tetramers or pentamers and cognate epitope stimulations. In agreement with the overlapping peptide stimulations, we found that resting HIV-SL9/IV9-tet+ cells showed increased expression of Eomes that was coupled to lower *ex vivo* expression of Granzyme B and perforin compared to CMV/NV9-tet+ cells. However, most HIV-SL9/IV9-tet+ cells were found to have high expression levels of Granzyme A (Figure S4A–B). Correlation analysis confirmed strong associations between the frequencies of cytolytic markers (perforin and Granzyme B) with T-bet/Eomes MFI in virus-specific tet+ cells (Figure S4C). Analysis on bulk CD8+ T cells further supported that perforin+ and Granzyme B+ cells were primarily T-bet^hi^ cells, while Granzyme A were expressed both within the T-bet^hi^ and Eomes^hi^ compartments (Figure S4D), thus clarifying the high Granzyme A content of HIV-tet+ cells. Cognate peptide stimulations additionally revealed that HIV-SL9/IV9-epitope specific CD8+ T cells, independently of whether they were bi- or monofunctional for IFNγ and/or CD107a, showed high expression levels of Eomes, but variable cytolytic content (Figure S4E). Interestingly, IFNγ+CD107a− epitope-specific cells showed increased signs of perforin, Granzyme B and Granzyme A expression compared to IFNγ-CD107a+ and IFNγ+CD107a+ cells (Figure S4F). These analyses further revealed that some HIV epitope-specific IFNγ-CD107a+ cells contained Granzyme A and B, but only in a minor fraction of the cells, which suggest that monofunctional CD107a+ cells might be highly exhausted (Figure S4E–F).

### Increased expression of inhibitory receptors and Eomes is traced to a transitional memory phenotype

**Figure 5:**
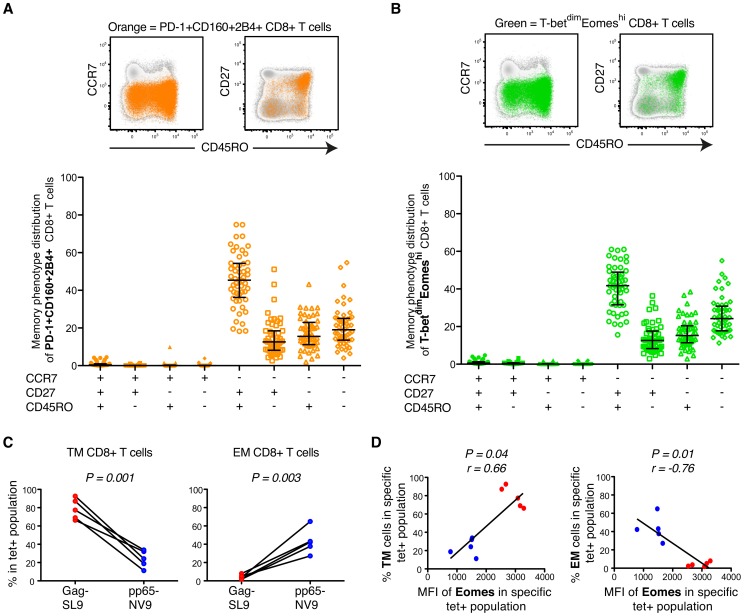
Phenotypic characterization of T-bet and Eomes expression in untreated HIV-infection. (A) Representative plots of an untreated HIV infected patient showing the distribution of total PD-1+CD160+2B4+ CD8+ T cells (orange) within different memory phenotype compartments, based on CD45RO, CD27 and CCR7 expression. The distribution of total PD-1+CD160+2B4+ CD8+ T cells was determined in all chronic untreated HIV infected subjects (B) FACS plots from an HIV infected subject showing the distribution of total T-bet^dim^Eomes^hi^ (green) cells within the different memory phenotype compartments. Also, the phenotypic distribution of total T-bet^dim^Eomes^hi^ CD8+ T cells within different memory compartments for all untreated HIV infected subjects. Median and IQR are shown for all populations. (C) The % of TM and EM compartmentalization for Gag/SL9-tet+ and pp65/NV9-tet+ cells. Paired t-tests were used to compare differences between the groups. (D) Correlations between the MFI of Eomes and TM or EM compartmentalization of Gag/SL9-tet+ and pp65/NV9-tet+ cells. Spearman non-parametric test was used to test for correlations.

We further traced the expression of the inhibitory receptors to diverse memory phenotypes using CD45RO, CD27 and CCR7 in the untreated HIV-infected subjects. The composition of bulk PD-1+CD160+2B4+ CD8+ T cells was particularly elevated within the transitional memory (TM; CD45RO+CD27+CCR7−) phenotype compartment (Figure 5A) as previously described [16]. Consistently, increased co-expression of the inhibitory receptors was associated with a higher frequency of TM cells, but not terminally-differentiated effector cells (Eff; CD45RO−CD27−CCR7−) (Figure S5A). PD-1+ and CD160+ cells were primarily found in the TM compartment, while 2B4+ cells were mainly effector memory (EM; CD45RO+CD27−CCR7−) and Eff cells. We next evaluated the phenotypic composition of T-bet and Eomes expressing cells and as expected found that T-bet^dim^Eomes^hi^ expressing cells were enriched and strongly associated with a transitional memory phenotype (Figure 5B and Figure S5B). Conversely, T-bet^hi^Eomes^dim^ expression was associated with increased EM (P = 0.032, r = 0.30) and particularly Eff (P<0.001, r = 0.69) cell compartmentalization (Figure S5C).

In agreement with other studies, we next confirmed that the majority of HIV-specific CD8+ T cells (median = 58%) accumulated in the TM compartment (Figure S5D) [26], [27]. CMV-specific CD8+ T cells on the other hand showed a balanced expression of T-bet and Eomes (Figure 2B) and were thus less encompassed to the TM compartment (Figure S5E). Similar to before, these results were further confirmed using HIV/SL9- and CMV/NV9-tetramers. The HIV/SL9-tet+ cells were indeed enriched within the TM compartment, while CMV/NV9-tet+ cells displayed a higher proportion of EM cells (Figure 5C). We next confirmed that expression levels of Eomes were positively associated (P = 0.04, r = 0.66) with the proportion of TM virus-specific CD8+ T cells, where as it was inversely correlated (P = 0.01, r = −0.76) with the frequency of EM virus-specific CD8+ T cells (Figure 5D). These data suggest that HIV-specific CD8+ T cell expression of numerous inhibitory receptors is linked to a transitional differentiation phenotype, displaying a T-bet^dim^Eomes^hi^ phenotype.

### Inflammatory cytokine levels are differentially associated with T-bet and Eomes expression

Inflammatory cytokines have previously been demonstrated to inversely regulate the expression levels of T-bet and Eomes in memory CD8+ T cells [5], [47], [48]. Therefore, we next aimed to determine whether the T-bet^dim^Eomes^hi^ or T-bet^hi^Eomes^dim^ expression profile of bulk CD8+ T cells was associated with the levels of IL-12p70, IFNα, TNF and IL-6 in untreated HIV infection (n = 38). Interestingly, we found tendencies of an inverse association pattern between T-bet and Eomes in terms of the inflammatory cytokine levels. While T-bet expression in general was positively associated with the cytokine plasma levels, Eomes instead was inversely associated with particularly the levels of IFNα (P = 0.015, r = −0.39) and TNF (P = 0.01, r = −0.41) (Figure S6). These data might implicate that bystander inflammation influence the bulk CD8+ T cell repertoire towards increased T-bet or Eomes expression in untreated HIV infection. HIV-specific CD8+ T cell compartmentalization within the T-bet^dim^Eomes^hi^ or T-bet^hi^Eomes^dim^ population was not associated with the levels of any inflammatory cytokine (data not shown).

### HIV-specific CD8+ T cells possess elevated expresion of inhibitory receptors and Eomes despite long-term ART

**Figure 6:**
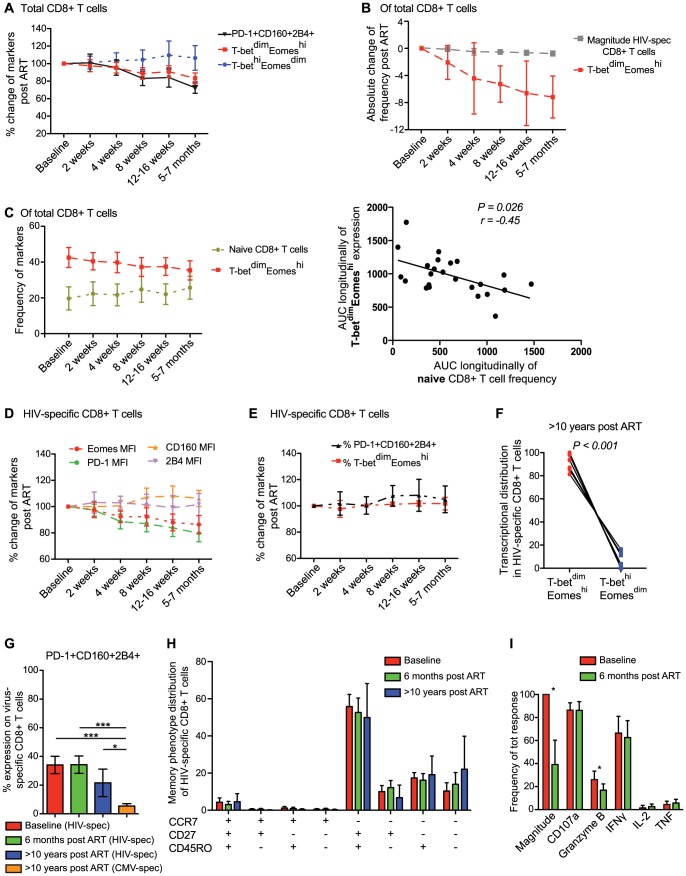
Longitudinal characterization of CD8+ T cell exhaustion and T-bet/Eomes expression following ART initiation. (A) Longitudinal analysis from baseline (same day as ART initiation) and 6 months post ART for total PD-1+CD160+2B4+ (black), T-bet^dim^Eomes^hi^ (red) and T-bet^hi^Eomes^dim^ (blue) expression on CD8+ T cells (n = 24). (B) The decay of HIV-specific CD8+ T cell magnitude (grey) and frequency T-bet^dim^Eomes^hi^ (red) post ART. (C) The frequency of naïve (asparagus) and T-bet^dim^Eomes^hi^ (red) expressing CD8+ T cells longitudinally post ART and Spearman correlation analysis between the area-under-curve (AUC) longitudinally for naïve and T-bet^dim^Eomes^hi^ expressing cells. Graphs illustrating the changes in frequency of (D) MFI of Eomes (red), PD-1 (green), CD160 (orange) and 2B4 (pink) expression, and (E) % of PD-1+CD160+2B4+ (black) and T-bet^dim^Eomes^hi^ (red) expression from baseline and longitudinally after 6 months on ART. All time-points represents the mean and 95% CI. (F) Distribution of HIV-specific CD8+ T cells within the T-bet^dim^Eomes^hi^ and T-bet^hi^Eomes^dim^ population after >10 years on ART (n = 12). (G) Frequency of HIV-specific PD-1+CD160+2B4+ CD8+ T cells at baseline (red), 6 months (green), >10 years on ART (blue) and CMV-specific PD-1+CD160+2B4+ CD8+ T cells >10 years on ART (orange). (H) Memory phenotype distribution of HIV-specific CD8+ T cells at baseline (red), 6 months (green) and >10 years on ART (blue). (I) Frequency of each individual function (of total HIV-specific response) before (red) and 6 months after ART (green) (n = 12). For G–I, median and IQR are provided for the bars, where (G–H) Kruskal Wallis test followed by Dunn's multiple comparison test was performed to compare 3 or more columns (***P<0.001, *P<0.05) and (I) Wilcoxon single rank tests (*P<0.05) were used for paired comparisons.

To determine whether the viral load had a direct effect on the expression patterns of Eomes, T-bet and the inhibitory receptors, we analyzed samples collected before and at 2, 4, 8, 12–16 weeks, and 5–7 months after ART initiation. These longitudinal analyses also allowed us to determine the kinetics of expression of these markers after succesful ART in 24 individuals. Before ART, the CD4 % was inversely associated (P = 0.015, r = −0.38) with the frequency of T-bet^dim^Eomes^hi^ cells (data not shown). Following initiation of ART, the frequency of T-bet^dim^Eomes^hi^ cells in total CD8+ T cells progressively declined in most individuals. In contrast, the frequency of T-bet^hi^Eomes^dim^ cells remained stable during the longitudinal assessment (Figure 6A). The decreased frequency of T-bet^dim^Eomes^hi^ cells was related to a similar decay of the PD-1+CD160+2B4+ population longitudinally after ART (Figure 6A), where area-under-curve measurements confirmed a close longitudinal relationship between these populations (P<0.001, r = 0.71; Figure S7A). We next assessed whether the decay of the T-bet^dim^Eomes^hi^ expressing CD8+ T cell population was a consequence of fading magnitudes of HIV-specific CD8+ T cells. However, the absolute decline of the T-bet^dim^Eomes^hi^ population was more pronounced (mean = 7.2%) than the loss of HIV-specific cells (mean = 0.76%; Figure 6B). Although the T-bet^dim^Eomes^hi^ population was associated (P = 0.003, r = 0.55) with the levels of CD8+ T cell activation (CD38+HLA-DR+) at baseline (Figure S7B), the decay of immune activation was not associated (P = 0.41, r = 0.18) with the fading T-bet^dim^Eomes^hi^ population longitudinally based on area-under-curve measurments (data not shown). Instead, we found that the decay of T-bet^dim^Eomes^hi^ cells was proportional to the recruitment of naïve CD8+ T cells after 6 months on ART (P = 0.026, r = −0.45; Figure 6C), indicating that the expression of Eomes in total CD8+ T cells might represent a balance between the frequency of naïve *versus* transitional memory CD8+ T cells.

We next analyzed the expression pattern of inhibitory receptors on the HIV-specific CD8+ T cells longitudinally after ART initiation. The MFI of CD160 and 2B4 remained stable after ART initiation, while expression levels of PD-1 declined in a hierarchical manner from baseline until 6 months post-ART initiation (P<0.001). The pattern of PD-1 expression on HIV-specific CD8+ T cells was related to the decay in Eomes MFI after ART initiation (Figure 6D). Despite this relationship between PD-1 and Eomes, we found no significant decline (P>0.05) in the co-expression of PD-1, CD160, and 2B4 on HIV-specific CD8+ T cells from before and 5–7 months after ART initiation (Figure 6E). This was linked to a steady T-bet^dim^Eomes^hi^ expression that persisted over the entire study period (Figure 6E). Notably, all individuals had fully suppressed viral load at 5–7 months, and in an attempt to clarify whether viremia had any effect on T-bet/Eomes or inhibitory receptors, we divided individuals into two groups based on whether the subjects had detectable or undetectable viremia after 12–16 weeks on ART. However, no significant differences were distingushiable between these groups in terms of HIV-specific T-bet^dim^Eomes^hi^ compartmentalization (P = 0.28) or PD-1, CD160, and 2B4 co-expression (P = 0.11; Figure S7C). Similarly, despite >10 years on ART (undetectable viral load for >8 years), the residual HIV-specific CD8+ T cells remained trapped within the T-bet^dim^Eomes^hi^ compartment (Figure 6F). Individuals on long-term ART showed tendencies of lower co-expression of the inhibitory receptors compared to individuals at baseline or on ART for 6 months (Figure 6G). However, the co-expression frequencies of the anti-CMV response for the long-term treated individuals was significantly lower than the anti-HIV response both pre- and post-ART (Figure 6G). Consistently, a majority of HIV-specific CD8+ T cell remained in the transitional memory compartment despite long-term therapy (Figure 6H) and showed similar functional characteristics, except increased Granzyme B expression pre-ART, before and after 6 months on ART (Figure 6I). These results together suggest that viral control by ART for several years does not change the phenotype of HIV-specific CD8+ T cells, which in turn might be due to retained elevated levels of Eomes expression.

## Discussion

T-bet and Eomes regulate the differentiation process of CD8+ T cells following encounter with a foreign antigen. Despite the central role of T-bet and Eomes in differentiation fate decisions, little is known about their influence on human CD8+ T cell responses [49]. Here, we show that human bulk and virus-specific CD8+ T cells expressing high levels of Eomes and low levels of T-bet, express several inhibitory receptors, display a transitional differentiation phenotype, exhibit poor functional abilities, and increased immune activation. This phenotype was unique to HIV-specific CD8+ T cells as compared to CMV-specific CD8+ T cells, and aid in explaining the HIV-specific CD8+ T cell subset's inability to clear or control the chronic viral replication.

Murine studies have demonstrated that T-bet and Eomes are centrally connected hub transcription factors that regulate exhaustion and memory fates of CD8+ T cells [28]. The negative regulation of inhibitory receptor expression by T-box transcription factors in CD8+ T cells was first demonstrated to involve direct binding of T-bet to the promotor region of PD-1, and increased expression of other inhibitory receptors, like CD160 and 2B4, in T-bet knock-out models [32]. Further analysis confirmed that T-bet deficiency in chronic LCMV infection also impact other neighbouring genes that are directly involved in CD8+ T cell differentiation and exhaustion, like Tigit, Itgam and IL-18Ra expression [28]. On the contrary, CD8+ T cell deficiency of Eomes leads to diminished PD-1 and Blimp-1 expression, as well as increased IFN-γ and TNF co-production following chronic LCMV infection, implicating that T-bet and Eomes regulate diverse sub-populations of CD8+ T cells [31]. In our study, T-bet and Eomes were expressed in distinct patterns and linked to the exhausted phenotype observed in the murine studies. The loss of polyfunctional HIV-specific CD8+ T cells was associated with increased expression levels of Eomes, and down-regulation of T-bet. These results confirm previous findings by Hersperger *et al*, demonstrating decreased expression levels of T-bet in chronic HIV progressors [33]. We also see tendencies that an increased fraction of viremic controllers (VL<1000) show increased signs of T-bet expression, but due to the low number of these subjects (n = 9), no significant correlations were detected between VL and T-bet/Eomes compartmentalization of HIV-specific CD8+ T cells in this cohort. Nevertheless, given that recent studies have shown that T-bet expression is co-localized both in the nucleus and cytoplasm [50], our results further suggest that HIV-specific CD8+ T cells in HIV chronic progressors might have high abundance of T-bet in the cytoplasm, leading to the inability to repress the expression of inhibitory receptors and induce cytolytic functions. However, a recent study by Ribeiro-dos-Santos *et al* concluded that loss of cytolytic potential of HIV-specific CD8+ T cells was a consequence of both T-bet and Eomes down-regulation in the chronic phase of HIV infection [51]. The discrepancies between this study and our results here are most likely due to the use of different techniques to observe the levels of transcription factors. While Ribeiro-dos-Santos *et al* isolated resting cell populations and used gene expression analysis to detect mRNA levels, we analyzed transcription factor protein expression on a single-cell level using flow cytometry. Both the peptide-stimulation protocol, as well as the tetramer staining for virus-specific T cells suggested that most HIV-specific CD8+ T cells had a T-bet^dim^Eomes^hi^ phenotype, indicating that stimulation with HIV antigens per se did not up-regulate Eomes expression in our analysis. Furthermore, immunoblot analysis have previously confirmed that T-bet and Eomes expression in the nucleus and cytoplasm resembles what is distinguishable with flow cytometry [50].

The lack of cytolytic functions of HIV-specific CD8+ T cells in chronic progressors has previously been observed in numerous studies [52], [53], [54]. Interestingly, we found a close correlation between the expression intensity of Eomes and monofunctionality measured as upregulation of CD107a. Although the CD107a single positive cells might represent highly exhausted cells, HIV-specific CD8+ T cells have been reported to contain high levels of Granzyme A [43], which partly was confirmed in our tetramer and cognate epitope stimulation assays. In addition, Eomes has been shown to also directly drive effector CD8+ T cell differentiation in T-bet knock-out mice [3] and therefore, we cannot conclude that Eomes high cells fail to deliver effector molecules. However, in experiments using effector (virus-specific) CD8+ T cells and target cells, the frequency of Granzyme B and perforin positive CD8 T cells is proportional to the percentage of target cells being lysed, while Granzyme A, -K and CD107a are not necessarily associated with specific lysis of cells [43], [52], [55]. T-bet has previously also been shown, in chromatin immunoprecipitation coupled with microarray analysis (ChIP-ChIP), to bind and promote increased gene expression of Granzyme B and perforin in T cells [56]. These data therefore suggest that HIV-specific CD8+ T cells in chronic progressors do not contain the proper cytotoxic granules to kill virus infected cells potentially due to poor up-regulation of T-bet.

The process of CD8+ T cell exhaustion is thought to be a consequence of increased antigen load, inflammation, and other events that drive the cells to an end-stage of their life cycle, where they lose the ability to induce effector functions. This hypothesis is supported by the fact that chronically high antigen levels cause T cell exhaustion during chronic viral infections [57]. In addition, HIV-infected individuals with low viral load generally have higher T cell polyfunctionality [25] and lower expression of inhibitory receptors on CD4+ T cells [58]. However, not all elite controllers show low expression of inhibitory receptors (unpublished observations) and despite viral control by ART, the functional chracteristics of HIV-specific CD8+ T cells are not fully restored [25], [53], [59]. Whether the state of exhaustion therefore is directly proportional to antigen burden and only due to chronicity is therefore not entirely clear. Instead, previous studies have shown that elevated HIV-specific CD8+ T cell expression of PD-1 occurs during early HIV infection and remains high in the chronic phase [60], possibly due to immune activation and the inability of CD8+ T cells to rest (M.R.B./M.B. unpublished observations). We, and others [61], have found associations between the state of immune activation (CD38+HLA-DR+) and Eomes, indicating that Eomes is up-regulated early after HIV infection as a consequence of immune activation, sustaining the expression of inhibitory receptors. However, monocyte activation in terms of sCD14 was not associated with increased expression of Eomes (data not shown), and no relationship between Eomes and CD38+HLA-DR+ CD8+ T cells was observed after ART initiation in longitudinal measurments. Whether the expression profile of increased Eomes and lower T-bet is a consequence, or cause, of chronic immune activation is therefore hard to determine. The associations between inflammatory cytokine levels and T-bet/Eomes expression, also suggest that the level of inflammation might influence the expression ratio between these transcription factors like previously observed in mice [5], [47], [48]. Nevertheless, our results strongly suggest at least that exhaustion of human viral-specific CD8+ T cells is not associated with T-bet up-regulation and thus terminal differentiation. These observations support previous studies showing a lack of association between markers of T cell exhaustion and terminal-differentiation (KLRG1 and CD57) in chronic LCMV and HIV infection [62], [63]. In agreement with these observations, our data suggest that less differentiated (TM) T cells show increased signs of exhaustion in comparison with EM T cells, possibly due to the reverse actions of T-bet and Eomes promoting diverse effector functions in the differentiation machinery. Further molecular delineation, and also identification of other transcription factors, that regulate T cell differentiation and exhaustion will therefore be informative for launching effective memory CD8+ T cell responses against persistent infections, like HIV, in future therapeutic settings.

Despite over 10 years on therapy, residual HIV-specific CD8+ T cells showed high expression levels of Eomes, inhibitory receptors and an intermediate differentiation status. The sustained co-expression of inhibitory receptors on HIV-specific CD8+ T cells was surprising as other studies have distinguished decreased PD-1 and CD160 expression after ART administration [16]. However, the longitudinal follow-up in this study was shorter (6 months post ART initiation) and after assesing the combined expression of PD-1, CD160, and 2B4 in individuals with >10 years on ART we found that these levels were lower. The longitudinal assessment of individuals initiating ART, nevertheless provided evidence that most HIV-specific CD8+ T cells showed elevated levels of inhibitory receptors and sustained expression levels of Eomes, compared to CMV-specific CD8+ T cells. Similarily, despite ART administration for 6 months, the HIV-infected subjects showed no significant improvement of functional characteristics compared to pre-ART. All of these individuals where however treated in chronic infection, and studies on patients initiating ART very early following infection would add valuable information on the dynamics of these responses. The persistent exhausted phenotype of virus-specific CD8+ T cells has also been observed in mice after antigen removal [64] and might be a consequence of unmethylated promotor regions of inhibitory pathways [65]. In addition to these data, our findings are supported by previous results showing that ART introduction or *in vitro* stimulations do not increase T-bet expression in HIV-specific CD8+ T cells [33], potentially through the reverse actions of Eomes. Altogether these data suggest that the exhausted phenotype is imprinted in HIV-specific CD8+ T cells during chronic infection, and remains stable several years with undetectable viral load.

In summary, we here show that HIV-specific CD8+ T cells retain an inverse expression pattern between T-bet and Eomes, which is highly associated with an exhausted phenotype. CMV-specific CD8+ T cells instead show increased signs of T-bet expression that potentially leads to the effector memory responses akin to those described by Hansen *et al* that control [66] or clear pathogenic SIV infection [67]. The sustained expression of Eomes and inhibitory receptors in/on HIV-specific CD8+ T cells after long-term ART further suggest that therapeutic strategies aimed at reinvigorating these responses might fail to elicit efficient responses to eradicate the viral reservoir. Future HIV vaccine or cure approaches most probably need to overcome this transcriptional barrier and induce sustained T-bet expression in order to clear virus infected cells.

## Supporting Information

Figure S1 **Expression patterns of inhibitory receptors and the Eomes/T-bet axis in healthy controls and HIV infected subjects.** (A) Representatives flow plots of the gating strategy to distinguish a pure CD8+ T cell population and the expression frequencies of all inhibitory receptors for total CD8+ T cells in one chronic untreated HIV infected subject, HIV+ (ART−) and one healthy control subject. (B) Representative patterns of T-bet and Eomes in conjunction with PD-1, CD160 and 2B4 expression on total CD8+ T cells in a HIV+ (ART−) subject. Correlations between the frequencies of T-bet^dim^Eomes^hi^ cells and PD-1, CD160, 2B4 and PD-1+CD160+2B4+ CD8+ T cells in all (C) long-term ART (ART+) treated HIV+ subjects and (D) healthy controls. Spearman non-parametric test was used to test for correlations.
(EPS)

Figure S2 **Median fluorescence intensity (MFI) expression of T-bet, Eomes and inhibitory receptors in/on virus-specific CD8+ T cells.** (A) The MFI expression of T-bet and Eomes between HIV- and CMV-specific CD8+ T cell responses. All data is derived from the untreated HIV infected subjects (n = 52). Median and IQR are shown in all graphs and non-parametric Mann-Whitney tests were performed to compare differences between the groups. (B) The MFI expression of PD-1, CD160, 2B4 and PD-1+CD160+2B4+ on HIV- and CMV-specific CD8+ T cells. Mann-Whitney tests were performed to conclude significance between the groups (median and IQR).
(EPS)

Figure S3 **Functional characteristics of CMV-specific CD8+ T cells in untreated HIV infection.** SPICE analysis of all functional combinations between the T-bet^dim^Eomes^hi^ (red) and T-bet^hi^Eomes^dim^ (blue) population for CMV-specific CD8+ T cells. Median and IQR are provided for all bars and whiskers. Wilcoxon matched-pairs single rank tests were performed to compare outcomes between groups; *P<0.05. Functional combinations where the IQR did not exceed 1% are not depicted in the graph. Permutation test was performed to test for functional diversity between the groups (pie charts).
(EPS)

Figure S4 **Tetramer analysis and cognate epitope stimulations for detection of virus-specific CD8+ T cell effector functions.** (A) The expression pattern of HIV-Gag/SL9-tet+ cells (red) between T-bet or Eomes (Y-axis) and perforin, Granzyme B or Granzyme A (X-axis) expression. (B) The frequency of perforin, granzyme B and Granzyme A expression for HIV-tet+ (red) and CMV-tet+ (blue) cells. Paired t-tests were used to compare differences between the groups. (C) Correlation analysis between the frequency of virus-specific perforin+, Granzyme B+ and Granzyme A+ cells and MFI of T-bet or Eomes. Spearman non-parametric test was used to test for significant correlations. (D) The relationship between T-bet and Eomes expression with perforin, Granzyme B and Granzyme A expression for resting CD8+ T cells. (E) Typical expression pattern of IFNγ+CD107a− (black), IFNγ+CD107a− (green) and IFNγ+CD107a+ (red) HIV epitope-specific cells for Eomes and Granzyme A expression. (F) Frequency (mean and 95% CI) of IFNγ+CD107a− (n = 5; black), IFNγ-CD107a+ (n = 5; green) and IFNγ+CD107a+ (n = 5; red) HIV epitope-specific cells expressing perforin, Granzyme B and Granzyme A. Paired t-tests showed no significant differences between the groups.
(EPS)

Figure S5 **Phenotypic associations of CD8+ T cells in untreated HIV infection.** (A) Correlation analysis between co-expression of PD-1, CD160 and 2B4 and the transitional memory (TM) or effector memory (Eff) CD8+ T cell compartment in all untreated HIV infected subjects (n = 52). Spearman non-parametric test was used to test for correlations. (B) Correlation between the frequency of T-bet^dim^Eomes^hi^ expressing cells and compartmentalization to the TM (CD45RO+CD27+CCR7−) phenotype (Spearman non-parametric test). (C) Association between the frequency of T-bet^hi^Eomes^dim^ expressing cells and Eff or effector memory (EM) compartmentalization (Spearman non-parametric test). Memory phenotype distribution of (D) HIV-specific (E) and CMV-specific CD8+ T cells, based on CD45RO, CD27 ad CCR7 expression. Median and IQR are shown for all populations.
(EPS)

Figure S6 **Cross-sectional correlations between inflammatory cytokines and T-bet/Eomes expression in untreated HIV infection.** Spearman correlation analyses between plasma levels (pg/mL) of IFNα (n = 38), IL-12p70 (n = 38), IL-6 (n = 38) and TNF (n = 38) and the frequencies of T-bet^dim^Eomes^hi^ or T-bet^hi^Eomes^dim^ expressing CD8+ T cells.
(EPS)

Figure S7 **Relationship between the longitudinal expression of T-bet/Eomes, inhibitory receptors and markers of immune activation.** (A) Area-under-curve measurement (AUC) of PD-1+CD160+2B4+ and T-bet^dim^Eomes^hi^ expressing CD8+ T cells longitudinally post ART, followed by Spearman non-parametric test to test for significant correlation. (B) Correlation between the frequency of T-bet^dim^Eomes^hi^ and CD38+HLA-DR+ expression on CD8+ T cells before ART initiation (Spearman non-parametric test). (C) Frequency of T-bet^dim^Eomes^hi^ and PD-1+CD160+2B4+ expressing CD8+ T cells between individuals with viral load >40 copies/mL (n = 8; black) and <40 copies/mL (n = 14; grey) 12–16 weeks post ART initiation. Mean and 95% CI are shown and based on the frequency of the markers after 12–16 weeks in comparison to baseline. Un-paired t-tests were performed to test for significance between the groups.
(EPS)

Table S1 **HIV RNA levels of longitudinal cohort.** Viral load and CD4 count measurements of the HIV-infected cohort (n = 24) that was longitudinally followed after ART initiation for 5–7 months.
(PDF)
