# Supplementary material for: T-bet and Eomes Are Differentially Linked to the Exhausted Phenotype of CD8+ T Cells in HIV Infection
Source: PLoS Pathog. 2014 Jul 17;10(7):e1004251. doi: 10.1371/journal.ppat.1004251 (PMC4102564; doi:10.1371/journal.ppat.1004251)
Supplement: Table S1 — HIV RNA levels of longitudinal cohort. Viral load and CD4 count measurements of the HIV-infected cohort (n = 24) that was longitudinally followed after ART initiation for 5–7 months. (PDF) [file ppat.1004251.s008.pdf]

Table S1

HIV RNA levels of longitudinal cohort

Time after ART initiation	HIV RNA, copies/mL	CD4 count, cells/µl
0 weeks	53000 (7960-208250)	330 (245-440)
2 weeks	585 (260-1550)	ND[A]
4 weeks	137 (44-475)	370 (330-446)
8 weeks	54 (40-150)	405 (330-553)
12-16 weeks	<40 (<40-63)	480 (330-610)
5-7 months	<40 (<40-<40)	490 (385-645)

Median (IQR) is shown for all parameters

[A] = Data points missing from >50% of cohort
